# Effect of Naringenin, Quercetin, and Sesamin on Xenobiotica-Metabolizing CYP1A and CYP3A in Mice Offspring after Maternal Exposure to Persistent Organic Pollutants

**DOI:** 10.1155/2017/8472312

**Published:** 2017-05-08

**Authors:** Nadezhda Pilipenko, Erik Ropstad, Ruth Halsne, Galia Zamaratskaia

**Affiliations:** ^1^Department of Molecular Sciences, Swedish University of Agricultural Sciences, P.O. Box 7015, 750 07 Uppsala, Sweden; ^2^Department of Production Animal Clinical Sciences, Faculty of Veterinary Medicine and Biosciences, Norwegian University of Life Sciences, P.O. 8146 Dep., 0033 Oslo, Norway; ^3^Faculty of Fisheries and Protection of Waters, South Bohemian Research Center of Aquaculture and Biodiversity of Hydrocenoses, University of South Bohemia in Ceske Budejovice, Zatisi 728/II, 389 25 Vodnany, Czech Republic

## Abstract

The aim of the present study was to evaluate in vitro effects of dietary phytochemicals naringenin, quercetin, and sesamin on the activities of ethoxy- (EROD; CYP1A) and benzyloxy- (BROD; CYP3A) resorufin O-dealkylases after the exposure to the cocktail of persistent organic pollutants (POPs). CD-1 mice were exposed from weaning, through gestation and lactation to a defined mixture of POPs. Hepatic microsomes were prepared from their female offspring at postnatal day 42. Hepatic EROD and BROD activity were evaluated in the presence of quercetin, naringenin, and sesamin at nine concentrations from 5 to 100000 nM. EROD activity was strongly inhibited by quercetin with *Ki* values from 1.7 to 2.6 *μ*M. BROD activity was inhibited by quercetin with *Ki* values from 64.9 to 75.3 *μ*M and naringenin with *Ki* values from 39.3 to 45.8 *μ*M. The IC_50_ and *Ki* values did not differ between the groups of mice with different levels of POPs exposure in any of the experimental sets. Sesamin did not inhibit either EROD or BROD. We concluded that the interactions of quercetin and naringenin with CYP1A and CYP3A in mice liver were not affected by the levels of POPs exposure.

## 1. Introduction

One of the greatest problems that the world is facing today is increased contamination of global environment by persistent organic pollutants (POPs). These compounds generally are not biodegradable and bioaccumulate in food chains, exerting adverse health effects in both humans and animals such as cancer risk, reproductive disorders, endocrine disruption, and genotoxicity [1].

Many pollutants act as inducers of several hepatic cytochrome P450 enzymes (CYP450) [1], which are responsible for metabolism of various endogenous compounds and xenobiotics. Thus, CYP450 activity can be used as a marker of contaminants in several species [2]. Among the CYP450s, CYP1A, CYP2E1, and CYP3A are involved in the activation of precarcinogens to highly reactive products which cause carcinogenicity in humans and experimental animals and thus are at least partly related to cancer formation [3, 4]. Some studies suggest that induction of catalytic activity of these enzymes is associated with increased risk of various forms of cancer (reviewed by [5, 6]). Humans are permanently exposed to several hundred anthropogenic chemicals at the same time, which are also known to modulate activities of CYP450 enzymes. Environmental pollutants often influence the expression of various genes by modulating inducible sequences in promoter regions, called responsive elements [6]. It is likely that organisms exposed to CYP450-inducing chemicals have an enhanced metabolism of drugs that are metabolized by the induced enzymes.

A number of plant-originated phytochemicals have been identified as inhibitors of CYP450 activity. These naturally occurring chemicals are usually associated with beneficial effects and low toxicity. Moreover, some studies demonstrated that they can be effective in cancer prevention [7]. For example, bergamottin, imperatorin, and isopimpinellin inhibited human CYP450 and block benzo[a]pyrene and 7,12-dimethylbenz[a]anthracene DNA adduct formation [8]. In mammals, quercetin and naringenin inhibited CYP1A and CYP3A activities in both in vivo and in vitro studies [9–12]. Sesamin, one of the major lignans from sesame seeds, inhibited human CYP2C9 [13] and fish CYP1A [14].

We hypothesized that induction of major drug-metabolizing enzyme activities by a mixture of POPs can be suppressed by phenolic compounds. Specifically, the aim of the present study was to evaluate in vitro effects of the dietary phytochemicals naringenin, quercetin, and sesamin on CYP1A and CYP3A activities in mice exposed to POPs. These two enzymes were chosen because of their importance in the metabolism of pollutants (especially CYP1A) and in drug metabolism (CYP3A).

## 2. Material and Methods

### 2.1. Animals and Sampling

Liver samples from CD-1 mice were obtained from the Section for Experimental Biomedicine at The Norwegian University of Life Sciences in Oslo, Norway. The unit is licensed by the Norwegian Food Safety Authority (NFSA; https://www.mattilsynet.no/dyr_og_dyrehold/dyrevelferd/forsoksdyr/) and accredited by the Association for Assessment and Accreditation of Laboratory Animal Care (https://www.aaalac.org). The study was approved by the unit's animal ethics committee (Institutional Animal Care and Use Committee/IACUC) and NSFA. The CD-1 F1 generation was exposed, through feed, to a defined mixture of POPs with the ratio of individual POP levels representing reported ratios in a Scandinavian diet. The exposure groups received either a low- or high-dose diet, estimated as 5000 times or 100,000 times human daily intake, respectively. Both control and exposure groups were fed from weaning, through gestation and lactation. The F2 generation, exposed in utero and during lactation, were euthanized by necropsy at postnatal day 42.

Liver samples were taken immediately after necropsy, frozen in ethanol and dry ice, and stored at −80°C until use. The microsomal fractions were prepared using a calcium aggregation method. Microsomal protein concentration was determined with a commercially available kit (Bio-Rad laboratories Inc., Hercules, CA, USA) according to the manufacturer's instructions.

### 2.2. CYP450 Activity Assays

In mice, O-dealkylation of 7-ethoxyresorufin is catalyzed by CYP1A1 and CYP1A2 [15], and O-dealkylation of 7-benzyloxyresorufin by CYP3A11 enzyme [16]. The activities of CYP1A and CYP3A were determined as a rate of ethoxy- (EROD) and benzyloxy- (BROD) resorufin O-dealkylation, respectively. Incubation mixtures contained microsomal protein (0.2 mg), phosphate buffer (pH 7.4, 50 mM), and appropriate substrate (1 *μ*M of 7-ethoxyresorufin or 2 *μ*M of 7-benzyloxyresorufin). Reactions were initiated by the addition of 0.5 mM NADPH. The reaction mixture, in a final volume of 500 *μ*L, was incubated in a water bath at 37°C for 5 min (EROD) or 7 min (BROD), and afterwards the reactions were terminated with ice-cold methanol (500 *μ*L), followed by centrifugation at 7,500 ×g. Resorufin concentrations were measured as previously described [11, 12]. EROD and BROD activities were expressed as pmol of resorufin per milligram protein and minute. Incubation conditions were linear with respect to incubation times and microsomal protein concentrations.

### 2.3. Inhibition Assays

EROD and BROD activities were initially evaluated in the presence of quercetin, naringenin, and sesamin at nine concentrations from 5 to 100000 nM. To examine the inhibition mode, the activities were determined in the presence of 2 or 3 inhibitor concentrations over the substrate concentration ranges 0.005 to 4.0 *μ*M for EROD and 0.05 to 8 *μ*M for BROD, respectively. The stock solutions of quercetin, naringenin, and sesamin were prepared in DMSO and added to incubations to yield final concentrations of 5, 10, 50, 100, 500, 1000, 2000, 1000, and 100000 nM. The final DMSO content in the incubations was 0.5%. The control incubations contained the same concentration of DMSO.

### 2.4. Data Analysis

Comparison of enzymatic activities in the absence of the inhibitors between studied groups was performed on logarithmically transformed values using a mixed model with fixed effect of treatment (SAS version 9.3, SAS Institute Inc., Cary, NC, USA). The IC_50_ (concentration causing 50% reduction of control activity) and *Ki* values for inhibitors were calculated by nonlinear regression analysis using GraphPad prism version 4.0 for Windows (GraphPad, San Diego, CA, USA). Comparisons of IC_50_ and *Ki* values between the studied groups were performed on logarithmically transformed values using one-way analysis of variance, followed by the Tukey multiple-comparisons test. Differences were regarded as significant when *P* < 0.05.

## 3. Results

The activities of EROD and BROD in mice without addition of phytochemicals differed and were highest in the offspring of mice that received the highest concentrations of POPs (Table 1).

**Figure 1:**
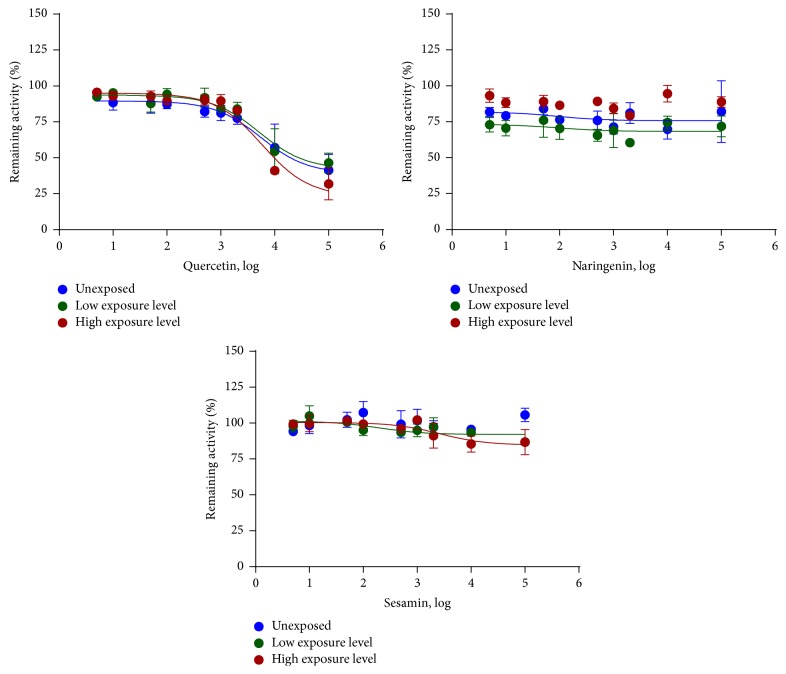
In vitro inhibition of CYP1A by quercetin, naringenin, and sesamin in the microsomes from mice exposed to different levels of POPs (*n* = 6 mice per group). CYP1A activity was measured by the rate of 7-ethoxyresorufin O-deethylation. Data are presented as the mean percentage of remaining activity and standard error of the enzyme activity in 6 mice.

**Figure 2:**
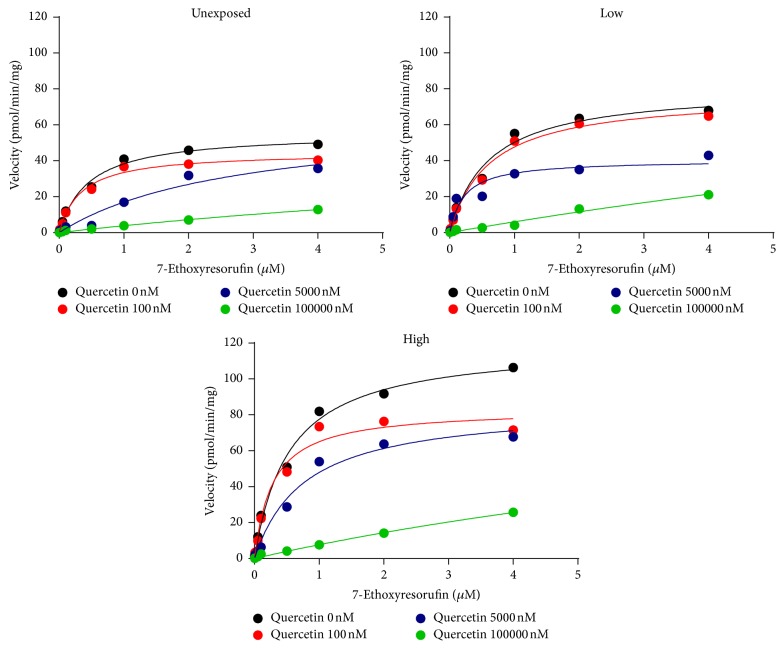
Michaels-Menten kinetics of 7-ethoxyresorufin O-dealkylation with or without quercetin in the microsomes from mice exposed to different levels of POPs.

EROD activity was competitively inhibited by quercetin (Figures 1 and 2; Table 2). Neither IC_50_ nor *Ki* values differed between the groups of mice with different levels of POPs exposure (Table 2). Neither naringenin nor sesamin inhibited EROD activity at the concentrations tested (Figure 1, Table 2).

**Figure 3:**
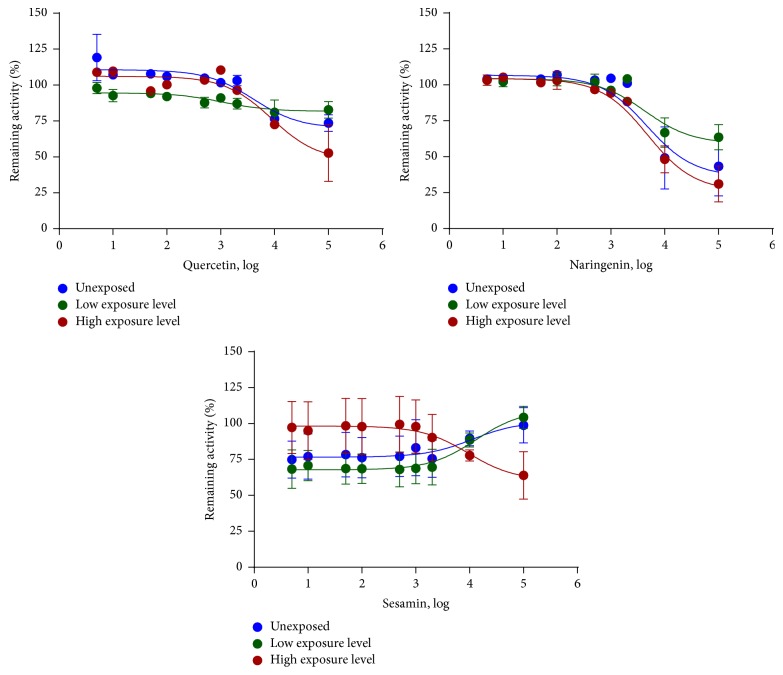
In vitro inhibition of CYP3A by quercetin, naringenin, and sesamin in hepatic microsomes from mice exposed to different levels of POPs (*n* = 6 mice per group). CYP3A activity was measured by the rate of 7-benzyloxyresorufin O-debenzylation. Data are presented as the mean percentage of remaining activity and standard error of the enzyme activity in 6 mice.

**Figure 4:**
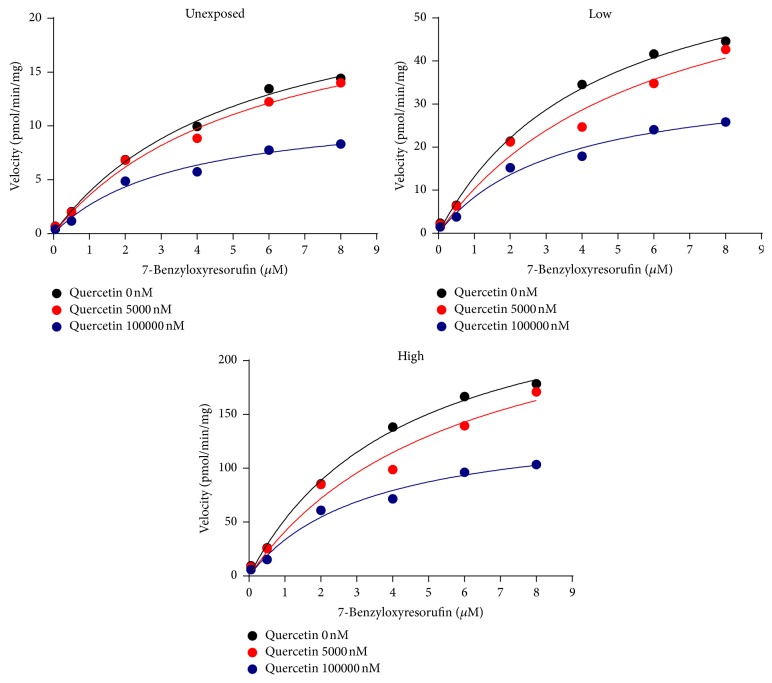
Michaels-Menten kinetics of 7-benzoxyresorufin O-dealkylation with or without quercetin in the microsomes from mice exposed to different levels of POPs.

**Figure 5:**
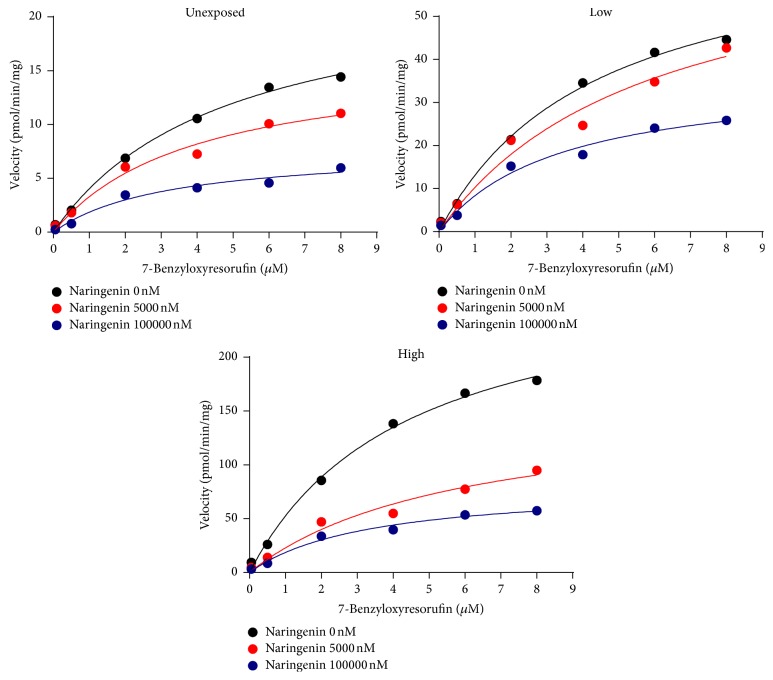
Michaels-Menten kinetics of 7-benzoxyresorufin O-dealkylation with or without naringenin in the microsomes from mice exposed to different levels of POPs.

BROD activity was noncompetitively inhibited by quercetin and naringenin (Figures 3, 4, and 5). The IC_50_ and *Ki* values of quercetin and naringenin were similar in all groups (Table 2). Sesamin did not inhibit BROD activity (Figure 3, Table 2).

## 4. Discussion

A wide array of plant-derived polyphenols decrease toxicant-induced oxidative stress and inflammation in various cell types, tissues, and animal species [17–20] and have been suggested to be protective against POP-mediated oxidative stress, inflammation, and toxicity [20]. It is likely that dietary flavonoids may play a role in protecting against POPs-related health disorders. Bearing in mind that CYP450 enzymes play an important role in carcinogenesis, identification of inhibitors of these enzymes is of huge interest.

A large number of studies investigated modulation of CYP450 activity by a single foreign compound, either drug, pollutant, or food-derived component [21]. However, exposure to a single compound does not reflect real life exposures. In the present study we investigated the ability of quercetin, naringenin, and sesamin to affect two major drug-metabolizing enzymes CYP1A and CYP3A after exposure to a mixture of POPs relevant to human exposure scenarios. Activity of both enzymes was previously shown to be induced by the presence of POPs [22]. It is well known that CYP1A-dependent EROD activity is induced by various toxicants including polycyclic aromatic hydrocarbons (PAH), polychlorated biphenyl (PCB), or polychlorodibenzo-p-dioxine (PCDD) [23–25]. As expected, EROD activity in the present study was higher in mice exposed to high POPs concentrations. Additionally, CYP1A activity is affected by the presence of bioactive compounds such as quercetin [26]. In the present study, the inhibitory potency of quercetin was compared in offspring of mice exposed to low and high levels of POPs. EROD activity was inhibited by quercetin with a similar magnitude in the control and exposed mice indicating that quercetin can interact with CYP1A and probably alter metabolism of CYP1A substrates independently of the levels of POPs exposure. Similarly, quercetin inhibited CYP3A-dependent BROD activity in mice independently of the levels of POPs exposure.

Naringenin inhibited CYP3A-dependent BROD activity somewhat stronger in the offspring of mice with high level of exposure of POPs. However, similarities in IC_50_ values imply no relevant effect of exposure to POPs on the ability of naringenin to affect CYP3A activity. Thus, we concluded that the interactions of quercetin and naringenin with CYP1A and CYP3A in mice liver were not affected by the levels of POPs exposure.

It should be emphasized that in vitro inhibition does not necessarily translate to physiologically relevant in vivo reduction of hepatic CYP1A activity. The fact that we used an in vitro approach in this study can be regarded as a limitation. Indeed, in vitro results cannot entirely reproduce a complex in vivo situation. However, we believe that in vitro studies provide a sound basis for in vivo approaches and is in agreement with a 3R (reduction, refinement, and replacement) strategy advocating use of a minimum number of animals.

In conclusion, the results of the present study demonstrated that the interactions of quercetin and naringenin with CYP1A and CYP3A in mice liver were not affected by the levels of POPs exposure.

## Figures and Tables

**Table 1:** Activities of CYP1A (EROD) and CYP3A (BROD) (pmol/min/mg pf protein) in the hepatic microsomes from mice of the unexposed, low-exposed, and high-exposed groups.

Enzyme	Level of exposure			*P* value
	Unexposed	Low	High	
CYP1A (EROD)	21.2^a^ ± 1.22	32.7^ab^ ± 1.22	46.5^b^ ± 1.22	0.078
CYP3A (BROD)	2.9^a^ ± 2.15	16.8^a^ ± 2.15	240.1^b^ ± 2.15	0.018

EROD, 7-ethoxyresorufin O-dealkylase; BROD, 7-benzyloxyresorufin O-dealkylase. The activities were measured using a single substrate concentration (1 *μ*M of 7-ethoxyresorufin for EROD, and 2 *µ*M of 7-benzyloxyresorufin for BROD). EROD and BROD activities were expressed as pmol of resorufin per minute and milligram protein. Data are presented as geometric means and standard errors. *P* value shows the overall effect of treatment on enzyme activity. Within a row mean values with different superscripts significantly differ (*P* < 0.05).

**Table 2:** IC_50_ and *Ki* values (*µ*M) for naringenin, quercetin, and sesamin calculated for mice from the unexposed, low-exposed, and high-exposed groups.

Enzyme	Phytochemical	Level of exposure			*P* value
		Unexposed	Low	High	
CYP1A (EROD)	Quercetin				
	IC_50_	5.4 (1.5–19.6)	5.1 (1.7–15.8)	5.8 (3.2–10.5)	0.819
	*Ki*	1.7 (1.2–2.3)	2.6 (1.9–3.4)	2.6 (1.8–3.2)	0.721
	Naringenin		No inhibition		
	Sesamin		No inhibition		

CYP3A (BROD)	Quercetin				
	IC_50_	30.2 (10.4–41.7)	27.1 (9.3–28.7)	35.7 (20.4–83.2)	0.266
	*Ki*	67.1 (50.3–83.7)	64.9 (34.3–76.3)	75.3 (29.1–93.8)	0.893
	Naringenin				
	IC_50_	40.3 (9.5–112.3)	49.1 (11.5–98.4)	43.1 (20.2–98.5)	0.925
	*Ki*	42.1 (30.3–78.9)	45.8 (25.6–82.1)	39.3 (28.5–64.9)	0.271
	Sesamin		No inhibition		

EROD, 7-ethoxyresorufin O-dealkylase; BROD, 7-benzyloxyresorufin O-dealkylase. Data are presented as geometric means and confidence interval in brackets. *P* value shows the overall effect of group on IC_50_ and *Ki* values.
